# Five-year trajectories of symptom severity, physical and mental functioning in patients with persistent somatic symptoms: the PROSPECTS cohort study

**DOI:** 10.1136/bmjopen-2023-083276

**Published:** 2025-01-08

**Authors:** Hieke Barends, Henriëtte E van der Horst, Johannes C van der Wouden, Nikki Claassen, Joost Dekker, Trynke Hoekstra

**Affiliations:** 1 Department of General Practice, Amsterdam UMC, Vrije Universiteit, Amsterdam, The Netherlands; 2 Amsterdam Public Health research institute, Amsterdam, The Netherlands; 3 Department of Rehabilitation Medicine and Department of Psychiatry, Amsterdam UMC, Vrije Universiteit, Amsterdam, The Netherlands; 4 Department of Epidemiology and Data Science, Amsterdam UMC, Amsterdam, The Netherlands; 5 Department of Health Sciences, Vrije Universiteit, Amsterdam, The Netherlands

**Keywords:** Prognosis, GENERAL MEDICINE (see Internal Medicine), Primary Care, Patient Reported Outcome Measures, Patient-Centered Care

## Abstract

**Abstract:**

**Objectives:**

Knowledge about the long-term course and prognosis of persistent somatic symptoms (PSS) is important to improve clinical decision-making and guidance for patients with PSS. Therefore, we aimed to: (1) identify distinct 5-year trajectories of symptom severity, physical and mental functioning in adult patients with PSS and (2) explore patient characteristics associated with these trajectories.

**Design:**

We used longitudinal data (seven measurements over a 5-year period) of the PROSPECTS study: a prospective cohort of adult patients with PSS. We applied Latent Class Growth Mixture Modelling to identify distinct trajectories for the three outcomes.

**Setting and participants:**

Patients were recruited in general practices and specialised treatment facilities for PSS throughout the Netherlands. The study population consisted of participants with three or more measurements available (n=297).

**Primary outcome measures:**

Symptom severity (Patient Health Questionnaire 15), physical and mental functioning (RAND-36 Physical Component Summary and Mental Component Summary).

**Results:**

For symptom severity, we identified two ‘stable’ trajectories: ‘severe symptoms, stable’ (15.8%) and ‘moderate symptoms, stable’ (84.2%). For physical functioning, we identified three trajectories: ‘poor physical functioning, marked improvement’ (8.5%); ‘poor physical functioning, stable’ (34.7%) and ‘moderate physical functioning, slight improvement’ (56.8%). For mental functioning, we identified three trajectories: ‘poor mental functioning, marked improvement’ (13.9%); ‘moderate mental functioning, deterioration’ (12.2%) and ‘moderate mental functioning, slight improvement’ (73.8%). Patients’ characteristics such as personal, social and environmental background, illness stressors, comorbid diseases, cognitive, emotional and behavioural responses varied for the distinct trajectories.

**Conclusions:**

We identified distinct 5-year trajectories for the three outcomes. Our findings suggest a high prevalence of persistence of symptoms and limited improvement in physical and mental functioning in the majority of patients with PSS. In a small proportion of patients, we identified trajectories that showed considerable physical or mental improvement or deterioration. Patient characteristics differed for the identified trajectories and may guide early recognition, although predictive studies are warranted.

**STRENGTHS AND LIMITATIONS OF THIS STUDY**

- Latent Class Growth Mixed Modelling (LCGMM)—a contemporary and flexible data-driven technique—was applied to identify distinct 5-year trajectories of symptom severity, physical and mental functioning in patients with persistent somatic symptoms (PSS).
- Strengths of this study include its multicentre prospective design, the long duration of follow-up using multiple measurements over time and that we assessed symptom severity (and persistence) as well as health-related functioning.
- The different trajectories found may adequately summarise general long-term trends, but we should keep in mind that LCGMM gives a simplified version of the complex heterogeneous reality.
- The majority of participants already experienced symptoms for an extensive period of time at inclusion (median duration 5 years; IQR 14 years), trajectories shortly after the onset of PSS thus may differ from the trajectories identified in this study.

## Introduction

 In primary care, up to one-third of patients present with somatic symptoms that cannot be (fully) explained by an organic disease.^1 2^ In specialist medical care, the numbers even run as high as 70%, depending on the specialty.^3 4^ When these symptoms persist, they are associated with substantial psychological distress and functional impairment.^5^,^7^ They are also a burden for society because of high healthcare use and work-related disability.^8 9^ There is ongoing debate about terms and definitions in this field. Throughout this manuscript, we will use the umbrella term ‘persistent somatic symptoms’ (PSS), see box 1 on the used definition.

Box 1 **Persistent somatic symptoms (PSS)**

Patients can suffer from PSS in the context of well-understood (adequately treated) conditions or when no somatic explanation for symptoms is found.^71 72^ Biological, psychological and social factors may all play a role in the persistence of somatic symptoms. In this study, PSS were defined as somatic symptoms that last at least several weeks and for which no sufficient somatic explanation is found after proper medical examination by a physician. This definition is in line with the Dutch guidelines for medically unexplained symptoms.^73 74^

The presentation and duration of PSS vary widely, from relatively mild and self-limiting symptoms at one end of the spectrum to persistent symptoms with severe impact on functioning at the other end. Knowledge about the long-term course and prognosis of PSS is scarce. Prior studies investigating the course of PSS have several shortcomings, for example, they are usually relatively limited in follow-up, use a single measurement to determine rates of improvement, worsening or chronicity and focus mainly on symptom persistence and not on physical and mental functioning, which are important aspects of prognosis for patients. A systematic review dating back to 2009 found that 50–75% of patients showed improvement in symptoms over time, while 10–30% worsened or became chronic.^10^ Most studies published after this review^11^,^18^ showed higher rates of symptom persistence, even after longer periods. For example, 56.8% of the participants had persistent symptoms after 2 years of follow-up in a primary care population.^15^ In sum, there is currently no study available that assessed long-term trajectories of PSS and most studies ignore physical and mental functioning.

Various patient characteristics have previously been associated with the prognosis of PSS,^10^,^20^ for example, sex^16 17 19^ and number of reported symptoms.^1011 13 15 18^,^20^ There is also a range of psychological factors that may influence the course of PSS.^21^ Until now, no study examined these characteristics in relation to different long-term trajectories of symptoms and functioning in patients with PSS.

The PROSPECTS cohort study on symptoms and physical and mental functioning in patients with PSS started in 2013.^22^ We have previously reported on the 2-year course of the PROSPECTS study.^23^ In the current publication, we focus on the 5-year course. We wanted to see if similar or new trajectories emerge over this longer period. In addition, we wanted to examine patient characteristics of the identified trajectories. To conclude, the aims of our current study are twofold: (1) to identify distinct trajectories of symptom severity, physical and mental functioning over a 5-year course in patients with PSS and (2) to explore patient characteristics associated with these trajectories.

## Method

### Context: the PROSPECTS study

**Figure 1:**
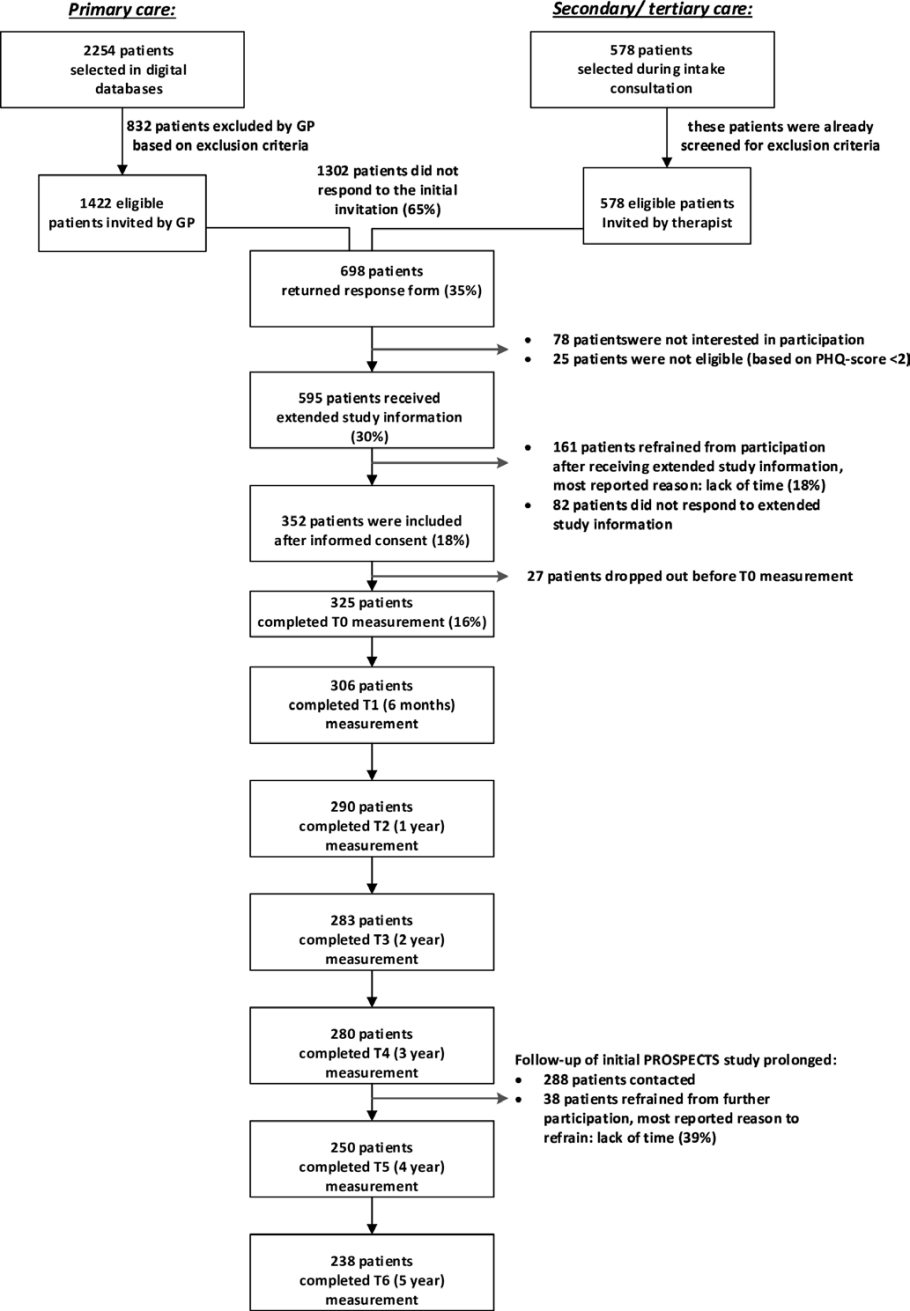
Flow chart of inclusion and longitudinal data collection of the PROSPECTS study. GP, general practitioner; PHQ, Patient Health Questionnaire.

The PROSPECTS study is a Dutch prospective cohort study on symptoms and physical and mental functioning in patients with PSS. A detailed research protocol and results of the recruitment process were published previously.^22 24^ In box 2 we present a summary of the patient selection procedure and in figure 1 a flow chart of the inclusion and follow-up of data collection of the PROSPECTS study. In total, 325 adult patients (18–70 years old) were recruited in general practices (n=218) and in specialised PSS programmes of secondary and tertiary care organisations (n=107) across the Netherlands in 2013–2015. Participants filled out questionnaires at predefined intervals. The questionnaires were sent by mail. The time intervals were kept constant. After the baseline measurement, follow-up measurements took place at 6 months, 1, 2, 3, 4 and 5 years. When participants missed one follow-up measurement, we offered them the option to resume participation at the next follow-up moment. As the study was extended after the first three follow-up years, written consent was again obtained before the 4-year measurement. Only participants who had not opted out of further participation were contacted.

Box 2 **Patient selection and flow chart PROSPECTS study**

**Patient selection**

In primary care, patients who visited their general practitioner (GP) ≥2 times with an unexplained physical symptom in the preceding 3 months were selected. An electronic database search was carried out, based on the ‘Robbins list’, a list of 23 frequently unexplained physical symptoms composed by Robbins *et al*,^82^ in combination with a lacking diagnosis International Classification of Primary Care (ICPC) code (meaning an ICPC code >70). Selected patients were checked for exclusion criteria by their own GP. In secondary and tertiary care, newly referred patients with medically unexplained symptoms as the reason for referral were screened for inclusion and exclusion criteria by the physician performing the intake consultation. The severity of symptoms was measured using the Patient Health Questionnaire 15 (PHQ-15).^25^ The patient had to have a score of 2 on at least one symptom of the PHQ-15 questionnaire, indicating that the symptom was bothering a lot, in order to participate in the PROSPECTS study.

Exclusion criteria were: (1) a sufficient medical explanation for the symptoms (according to the physician), (2) incomplete diagnostic evaluation of the symptoms (according to the physician), (3) insufficient command of the Dutch language, (4) a cognitive or visual impairment that prohibited participating in a questionnaire survey, (5) severe psychopathology (eg, psychotic disorder, bipolar disorder), (6) pregnancy, (7) cancer diagnosed in the 5 years prior to inclusion or (8) another life-threatening condition or a short life expectancy.

### Patient and public involvement

Patients or the public were not actively involved in the design, conduct, reporting or dissemination plans of our research.

### Study population

For the present study, we used seven measurements in time (baseline, 6 months, 1, 2, 3, 4 and 5 years data). For this analysis, we excluded participants (n=3) in whom their PSS turned out to be fully explained by a somatic disease that was diagnosed during follow-up (pancreas carcinoma, congenital hip dysplasia, Crohn’s disease). Participants with a minimum of three measurements available were selected as the study population for this analysis (n=297; n=196 recruited in primary care; n=101 recruited in specialised PSS care). Measurements of participants with a newly diagnosed serious disease during follow-up (such as a life-threatening cancer diagnosis) were censored from the time of disease onset.

### Measures

The primary outcomes of the trajectories were symptom severity (Patient Health Questionnaire-15 (PHQ-15)), physical functioning (RAND-36 Physical Component Summary (PCS)) and mental functioning (RAND-36 Mental Component Summary (MCS)) collected at 0, 6 months, 1, 2, 3, 4 and 5 years of follow-up (see online supplemental appendix A).

#### Symptom severity (primary outcome)

The 15-item PHQ-15^25^ was used as an indicator of the level of somatic symptom severity. The PHQ-15 is a reliable measure of somatic symptom reporting in this field of research.^26^,^28^ A higher score on the scale indicates more severe symptoms. We considered a change of at least five points a minimal important change (MIC) on the PHQ-15. Based on priory suggested cut-offs points, we considered a score below 5 as ‘minimal’, a score of 5–9 as ‘mild’, a score of 10–14 as ‘moderate’ and a score of 15 or higher as ‘severe’ somatic symptoms.^25^

#### Physical and mental functioning (primary outcomes)

The PCS and MCS of the RAND-36 questionnaire (V.2.0) were used as indicators of physical and mental functioning.^29 30^ The RAND-36 questionnaire is a 36-item self-report survey of patient health in the preceding month. It was selected because of its validity^30^ and previous use in this area of research. The RAND-36 PCS and RAND-36 MCS were calculated to summarise physical and mental functioning into a 0–100 scale score. A higher score indicates better functioning. A change of at least 0.5 times the SD (10 points on both scales in this study) for physical and mental functioning is considered an MIC.^31^ We considered a score above 70 as ‘average’, between 40–70 as ‘moderate’ and below 40 as ‘poor’ physical and/or mental functioning.

#### Patient characteristics associated with trajectories

We explored the association of several patient characteristics for the identified trajectories of PSS. The selection of patient characteristics and questionnaires (see online supplemental appendix A) was based on characteristics previously associated with the prognosis of PSS,^10^,^20^ theoretical models^32^ and availability in our data set.^22^ Patient characteristics were measured partly at baseline (T0) and partly at the first follow-up measurement at 6 months (T1), in order to reduce participant burden at baseline; the characteristics measured at T1 are considered relatively stable over time in adults.^33^ For clarity, patient characteristics were categorised into categories suggested in previous literature:^34^ personal background, social and environmental background, illness stressors, comorbid diseases and cognitive, emotional and behavioural responses.

### Statistical analysis

Data analyses were performed using IBM (New York, USA) SPSS Statistics (V.26) and Muthén & Muthén (Los Angeles, USA) Mplus (V.8.5). Descriptive statistics are presented as mean with SD for normally distributed continuous data; median, IQR for skewed continuous variables and as numbers and percentages for dichotomous and categorical variables.

#### Identifying distinct trajectories by Latent Class Growth Mixture Modelling

In Latent Class Growth Mixture Modelling (LCGMM), the assumption is that within a study sample, individuals may come from distinct underlying (or latent) subpopulations, that follow different and unique trajectories over time. The main aim is to identify the number and characteristics of these trajectories.

A ‘forward approach’ was applied,^35^ similar to that in the analyses of the 2-year course of the PROSPECTS study,^23^ starting with a model with one trajectory and adding more trajectories while evaluating the model fit. Models with one to six trajectories were run. We determined the optimal number of trajectories by using several statistical parameters combined with the clinical interpretability of the identified trajectories, in order to avoid clinically uninterpretable trajectories.^35^ Statistical parameters included the Bayesian information criterion (BIC), the Akaike information criteria (AIC), posterior probabilities and entropy.^36^ Both a lower BIC and AIC correspond to a better model fit.^36 37^ In addition, the higher the posterior probabilities and the higher the entropy (closer to 1), the fewer the classification errors and the less bias in the prediction of trajectory membership.^38^ Posterior probabilities of at least 0.7 or 0.8 are advised.^39^,^41^ Entropy indicates the ‘fuzziness’ of the model and is a common indicator of uncertainty in classification.^41 42^

Unequally spaced time points of our data collection (ie, intervals of 6 months and 12 months) were modelled. As we anticipated within-class heterogeneity based on the 2-year analyses,^23^ we used LCGMM instead of Latent Class Growth Analysis.^43 44^ LCGMM allows for variation in intercept and slope in one or more classes, leading to larger within-class heterogeneity.^35 41 45^ To accommodate anticipated fluctuations over time based on the 2-year analyses,^23^ we estimated and compared models with linear and quadratic terms. In LCGMM, missing data are handled with the Expectation-Maximation Algorithm^46^; therefore, there was no need to impute missing data. Trajectories resulting from the LCGMM were visualised alongside the mean trajectories of all participants. Subsequently, inter-individual and intra-individual variation within the different trajectories was visualised by producing graphs containing the individual courses of all participants per identified trajectory.

#### Differences in patient characteristics for the identified trajectories

We followed a standard three-step method as described by Andersen *et al*.^47^ First, we determined the optimal number of latent trajectories as described above (step 1); subsequently, we selected the most likely trajectory for each participant (step 2) and finally, we explored differences in the selected patient characteristics between the identified trajectories (step 3).

For the selected patient characteristics, the questionnaires used^2529 30 48^,^68^ can be found in online supplemental appendix A. Normally distributed data were tested with parametric tests; non-parametric tests were used for non-normally distributed data. For categorical variables, we used χ^2^ tests. For continuous variables with a normal distribution we used either the (parametric) independent sample t-tests or analysis of variance and post hoc Bonferroni correction, depending on the number of trajectories identified. Post hoc Bonferroni correction was applied to control for the overall probability of type I error (false positive) for multiple hypothesis testing. For continuous variables with skewed distribution, we used the (non-parametric) Mann-Whitney test or Kruskal-Wallis test, depending on the number of trajectories.

## Results

### Study population

Characteristics of the study population at the baseline measurement are presented intable 1. At recruitment, participants showed on average moderate levels of somatic symptoms severity and moderate levels of physical and mental functioning. The three most frequently reported somatic comorbid diseases were ‘osteoarthritis’ (21.9%, n=65), ‘hypertension’ (19.5%, n=58) and ‘eczema or other skin disease’ (16.5%, n=49). The three most frequently mentioned psychiatric comorbid disorders were ‘burn-out syndrome’ (25.3%, n=75), ‘depressive disorder’ (18.2%, n=54) and ‘anxiety disorder’ (15.2%, n=45). The mean duration of symptoms at recruitment was 9.4 years (SD 11.1 years) and the median duration was 5 years (IQR 14 years), indicating that the distribution was skewed to the right. Based on cut-offs presented in prior literature, the study population scored on average mild on both anxiety and depression severity^69^ and high on health anxiety^70^ (table 1).

**Table 1:** Characteristics of the study population (n=297) at baseline

	Study population (N=297)	N missing (%)
**Outcomes**		
Symptom severity (PHQ-15, scale 0–30), mean (SD)	12.3 (5.3)	0 (0)
Physical functioning (RAND-36 PCS, scale 0–100), mean (SD)	47.1 (19.5)	4 (1.3)
Mental functioning (RAND-36 MCS, scale 0–100), mean (SD)	53.0 (20.2)	4 (1.3)
**Personal background**		
Women, n (%)	226 (76.1)	0 (0)
Education level, n (%)		0 (0)
Lower education	98 (33.0)	
Intermediate education	117 (39.4)	
Higher education	82 (27.6)	
Age in years, range 19–70, mean (SD)	46.8 (12.4)	0 (0)
Potentially traumatic events in childhood (<16 years) (LEQ), median (IQR; total range)	0 (1; 0–7)	0 (0)
Perfectionism (MDPS, scale 29–145), mean (SD)*	70.1 (22.6)	30 (10.1)
Neuroticism (NEO-FFI, scale 12–60), mean (SD)*	32.6 (9.0)	16 (5.4)
Extraversion (NEO-FFI, scale 12–60), mean (SD)*	38.4 (6.5)	17 (5.7)
**Social and environmental background**		
Marital status, n (%)		0 (0)
Married or cohabiting	181 (60.9)	
Other (single, divorced, widow)	116 (39.1)	
Social support (SoS, scale 12–60), median (IQR; total range)	56 (12; 15–60)	0 (0)
**Illness stressors**		
Number of symptoms (PSQ), mean (SD)	22.5 (9.5)	2 (0.7)
Duration of symptoms in years, median (IQR; total range)	5 (14; 0.02–62)	8 (2.7)
**Comorbid diseases**		
Number of somatic comorbid diseases (TiC-P), median (IQR; total range)	1 (2; 0–8)	2 (0.7)
Number of psychiatric comorbid disorders (TiC-P), median (IQR; total range)	0 (1; 0–3)	2 (0.7)
**Cognitive responses**		
Fear-avoidance cognitions (CBRQ, scale 0–24), mean (SD)	9.9 (4.7)	3 (1.0)
Catastrophising cognitions (CBRQ, scale 0–16), mean (SD)	5.7 (3.5)	2 (0.7)
Damage cognitions (CBRQ, scale 0–20), mean (SD)	9.8 (3.6)	3 (1.0)
Embarrassment avoidance cognitions (CBRQ, scale 0–24), median (IQR; total range)	7.0 (8; 0–24)	3 (1.0)
Symptom focusing (CBRQ, scale 0–24), mean (SD)	10.4 (5.2)	5 (1.7)
Somatosensory amplification (SSAS, scale 10–50), mean (SD)	27.1 (6.3)	1 (0.3)
Identity (label of disease and associated symptoms, IPQ-brief, scale 0–10), mean (SD)	7.2 (1.9)	0 (0)
Consequences of illness (IPQ-brief, scale 0–10), median (IQR; total range)	8 (3.5; 0–10)	0 (0)
Timeline (expected duration of illness, IPQ-brief, scale 0–10), median (IQR; total range)	8 (4; 0–10)	1 (0.3)
Personal control (over illness, IPQ-brief, scale 0–10), mean (SD)	5.9 (2.8)	0 (0)
Treatment control (beliefs about treatment effect on illness, IPQ-brief, scale 0–10), mean (SD)	4.3 (2.6)	4 (1.3)
Comprehension of illness (extent to which patients believe to understand their illness, IPQ-brief, scale 0–10), mean (SD)	4.1 (2.7)	0 (0)
**Emotional responses**		
Anxiety severity (BAI, scale 0–63), median (IQR; total range)	10 (11.3; 0–51)	19 (6.4)
Depression severity (QIDS-SR, scale 0–27), mean (SD)	9.2 (5.0)	3 (1.0)
Positive affect (PANAS, scale 10–50), mean (SD)	32.5 (8.2)	4 (1.3)
Health anxiety (WI, scale 0–14), mean (SD)	5.8 (2.6)	16 (5.4)
Concern (about illness, IPQ-brief, scale 0–10), mean (SD)	5.5 (2.8)	1 (0.3)
Emotions (extent to which illness affects mood, IPQ-brief, scale 0–10), mean (SD)	7 (2.6)	0 (0)
**Behaviouralresponses**		
All-or-nothing behaviour (CBRQ, scale 0–20), mean (SD)	7.8 (4.5)	4 (1.3)
Avoidance/resting behaviour (CBRQ, scale 0–32), mean (SD)	8.2 (4.8)	11 (3.7)
Physical activity (IPAQ, total METs per week), median (IQR; total range)	2729 (5832;0–63 312)	18 (6.1)

* As indicated in the method section, these characteristics were measured at T1.

BAIBeck Anxiety InventoryCBRQCognitive Behavioural Response QuestionnaireIPAQInternational Physical Activity QuestionnaireIPQ-briefbrief illness perception questionnaireLEQLife Events QuestionnaireMDPSMultidimensional Perfectionism ScaleMETsMetabolic EquivalentsNEO-FFINEO Personality Questionnaire-Five Factor InventoryPANASsubscale of Positive and Negative Affect SchedulePHQ-1515-item Patient Health QuestionnairePSQPhysical Symptom QuestionnaireQIDS-SRQuick Inventory of Depressive SymptomatologyRAND-36RAND 36-item Health SurveyRAND-36 MCSRAND-36 Mental Component SummaryRAND-36 PCSRAND-36 Physical Component SummarySoSSocial Support ScaleSSASSomatosensory Amplification ScaleTiC-PTreatment Inventory of Costs in patients with psychiatric disordersWIWhitely Index

### Results of LCGMM

Of the 297 participants, 217 (73%) completed all seven measurements. Overall, quadratic models with estimated within-class intercept variance performed best, these models are presented here (for model fit indices, see online supplemental appendix B).

#### Trajectories of symptom severity

**Figure 2:**
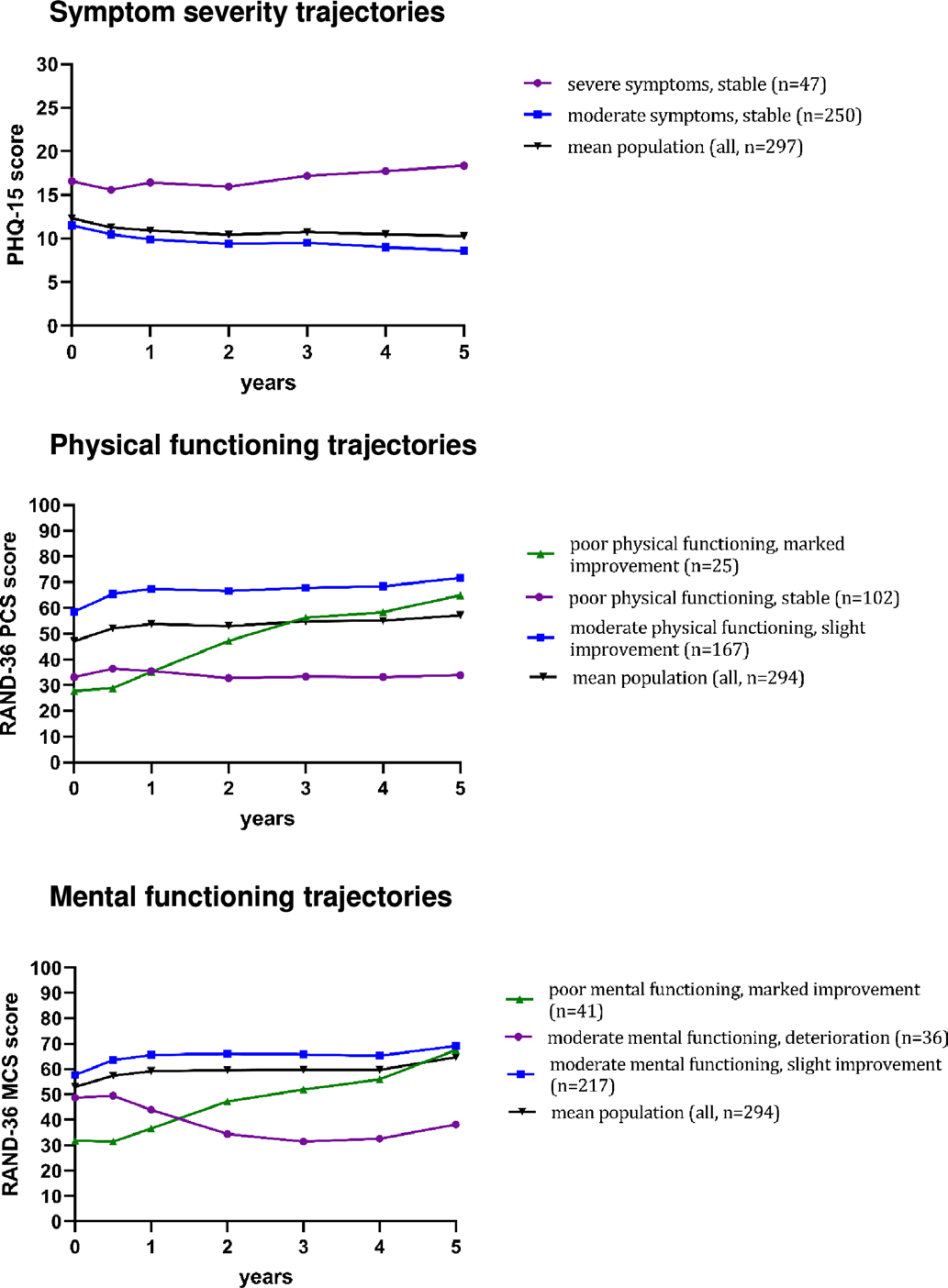
Identified 5-year trajectories. PHQ-15, Patient Health Questionnaire-15; RAND-36 MCS, RAND-36 Mental Component Summary; RAND-36 PCS, RAND-36 Physical Component Summary.

We took into account BIC, posterior probabilities, entropy, class sample size as well as clinical interpretability. This resulted in the selection of the two-trajectory model (see figure 2), containing a ‘severe symptoms, stable’ (15.8%, n=47) and a ‘moderate symptoms, stable’ (84.2%, n=250) trajectory. Based on the MIC for the PHQ-15 (5-point change), both trajectories showed no clinically relevant improvement or deterioration over 5 years. Note that the posterior probability of the ‘stable moderate symptoms’ trajectory was suboptimal (0.618, ie, below the desired >0.7), indicating that for this trajectory there was more uncertainty in classification.

#### Trajectories of physical functioning

Model fit indices for the physical functioning trajectories are presented in online supplemental appendix B. Models with 3-trajectories and 4-trajectories were close in BIC, AIC was slightly better for the 4-trajectory model, however, adding an additional trajectory did not result in a clinically useful additional cluster. As posterior probabilities and entropy were acceptable for the 3-trajectory model, this model was selected (see figure 2), consisting of a ‘poor physical functioning, marked improvement’ (8.5%, n=25) trajectory, a ‘poor physical functioning, stable’ (34.7%, n=102) trajectory and a ‘moderate physical functioning, slight improvement’ (56.8%, n=167) trajectory. Based on the MIC for the RAND-36 PCS (10-point change), the ‘poor physical functioning, marked improvement’ trajectory showed clear clinically relevant improvement over 5 years (about four times the MIC). The ‘moderate physical functioning, slight improvement’ trajectory improved by about the MIC over the 5-year course.

#### Trajectories of mental functioning

Based on the combination of fit indices, class sample size as well and clinical interpretability we selected the 3-trajectory model (see figure 2), including a ‘poor mental functioning, marked improvement’ (13.9%, n=41) trajectory, a ‘moderate mental functioning, deterioration’ (12.2%, n=36) trajectory and a ‘moderate mental functioning, slight improvement’ (73.8%, n=217) trajectory. For the ‘poor mental functioning, marked improvement’ trajectory there was more uncertainty in classification (posterior probability below the desired >0.7, see online supplemental appendix B). Based on the MIC for the RAND-36 MCS (10-point change), the ‘poor mental functioning, marked improvement’ trajectory improved roughly 3.5 times the MIC over the course of 5 years. For the ‘moderate mental functioning, slight improvement’ trajectory, participants improved by about the MIC. Participants in the ‘moderate mental functioning, deterioration’ trajectory deteriorated by on average twice the MIC.

#### Visual evaluation of identified trajectories

We produced graphs for individual participants per trajectory (see online supplemental appendix C). Visual inspection of these graphs indicated considerable inter-individual and intra-individual variation in the identified trajectories.

### Exploring patient characteristics for identified trajectories

#### Patient characteristics for trajectories of symptom severity

The results of the analyses are presented in table 2. Patients in the ‘severe symptoms, stable’ trajectory scored lower on extraversion, were less often married or cohabiting and reported lower social support. They reported a higher number of symptoms and more somatic comorbid diseases at recruitment. In addition, they reported more unfavourable cognitive, emotional and behavioural responses than patients in the ‘moderate symptoms, stable’ trajectory.

**Table 2:** Differences in patient characteristics for trajectories of symptom severity

Symptom severity (PHQ-15) trajectories			
Trajectory	(A) Severe symptoms, stable (n=47)	(B) Moderate symptoms, stable (n=250)	P value between clusters
**Personal background**			
Women, n (%)	41 (87.2)	185 (74.0)	p=0.051
Education level, n (%)			p=0.222
Lower education	20 (42.6)	78 (31.2)	
Intermediate education	18 (38.3)	99 (39.6)	
Higher education	9 (19.1)	73 (29.2)	
Age in years, range 19–70, mean (SD)	47.1 (11.6)	46.7 (12.5)	p=0.830
Potentially traumatic events in childhood (<16 years) (LEQ), median (IQR; total range)	1 (2; 0–6)	0 (1; 0–7)	p=0.073
Perfectionism (MDPS), mean (SD)*	71.5 (24.5)	69.9 (22.3)	p=0.669
Neuroticism (NEO-FFI), mean (SD)*	35.0 (7.8)	32.2 (9.1)	p=0.056
Extraversion (NEO-FFI), mean (SD)*	36.6 (6.7)	38.7 (6.4)	**p=0.044**
**Social and environmental background**			
Marital status, n (%)			**p=0.030**
Married or cohabiting	22 (46.8)	159 (63.6)	
Other (single, divorced, widow)	25 (53.2)	91 (36.4)	
Social support (SoS), median (IQR; total range)	53 (14; 22–60)	57 (11; 15–60)	**p=0.004**
**Illness stressors**			
Number of symptoms (PSQ), mean (SD)	30.8 (7.6)	20.9 (9.0)	**p<0.001**
Duration of symptoms in years, median (IQR; total range)	10 (19; 0.06–45)	4 (13; 0.02–62)	p=0.096
**Comorbid diseases**			
Number of somatic comorbid diseases (TiC-P), median (IQR; total range)	2 (2; 0–8)	1 (2; 0–7)	p<0.001
Number of psychiatric comorbid disorders (TiC-P), median (IQR; total range)	0 (1; 0–3)	0 (1; 0–3)	p=0.312
**Cognitive responses**			
Fear-avoidance cognitions (CBRQ), mean (SD)	11.9 (4.7)	9.5 (4.7)	**p=0.002**
Catastrophising cognitions (CBRQ), mean (SD)	6.9 (3.2)	5.4 (3.5)	**p=0.009**
Damage cognitions (CBRQ), mean (SD)	10.7 (3.7)	9.6 (3.6)	p=0.069
Embarrassment avoidance cognitions (CBRQ), median (IQR; total range)	10 (7; 1–22)	6 (9; 0–24)	**p<0.001**
Symptom focusing (CBRQ), mean (SD)	10.8 (5.2)	10.3 (5.2)	p=0.62
Somatosensory amplification (SSAS), mean (SD)	29.8 (5.8)	26.6 (6.2)	**p=0.001**
Identity (label of disease and associated symptoms, IPQ-brief), mean (SD)	8.2 (1.6)	7.0 (1.9)	**p<0.001**
Consequences (beliefs about illness effects and outcomes, IPQ-brief), median (IQR; total range)	9 (3; 2–10)	7 (4; 0–10)	**p<0.001**
Timeline (expected duration of illness, IPQ-brief), median (IQR; total range)	9 (3; 0–10)	8 (4; 0–10)	p=0.257
Personal control (over illness, IPQ-brief), mean (SD)	6.0 (2.9)	5.9 (2.8)	p=0.700
Treatment control (beliefs about treatment effect on illness, IPQ-brief), mean (SD)	4.9 (2.9)	4.2 (2.5)	p=0.121
Comprehensibility (extent to which patients believe to understand their illness, IPQ-brief), mean (SD)	3.8 (2.4)	4.1 (2.8)	p=0.465
**Emotional responses**			
Anxiety severity (BAI), median (IQR; total range)	17 (18; 6–44)	9 (10; 0–51)	**p<0.001**
Depression severity (QIDS-SR), mean (SD)	12.2 (4.6)	8.6 (4.6)	**p<0.001**
Positive affect (PANAS), mean (SD)	29.7 (7.1)	33.0 (8.3)	**p=0.010**
Health anxiety (WI), mean (SD)	7.3 (3.0)	5.6 (2.6)	**p<0.001**
Concern (about illness, IPQ-brief), mean (SD)	6.2 (2.9)	5.4 (2.8)	p=0.070
Emotions (extent to which illness affects mood, IPQ-brief), mean (SD)	7.4 (2.5)	6.0 (2.9)	**p=0.003**
**Behaviouralresponses**			
All-or-nothing behaviour (CBRQ), mean (SD)	9.8 (4.0)	7.5 (4.5)	**p=0.001**
Avoidance/resting behaviour (CBRQ), mean (SD)	10.3 (4.6)	7.8 (4.8)	**p=0.002**
Physical activity (IPAQ, in METs), median (IQR; total range)	1916 (3142; 0–44 436)	2880 (6703; 0–63 312)	p=0.087

In bold p<0.05.

* As indicated in the method section, these characteristics were measured at T1 (6 months follow-up); differences between trajectories were tested with independent sample t-test for mean values, with χ2 tests for proportions and Mann-Whitney U test for median values.

BAIBeck Anxiety InventoryCBRQCognitive Behavioural Response QuestionnaireIPAQInternational Physical Activity QuestionnaireIPQ-briefBrief Illness Perception Questionnaire (higher scores indicating more negative illness perceptions)LEQLife Events QuestionnaireMDPSMultidimensional Perfectionism ScaleMETsMetabolic EquivalentsNEO-FFINEO Personality Questionnaire-Five Factor InventoryPHQ-15Patient Health Questionnaire-15PSQPhysical Symptom QuestionnaireQIDS-SRQuick Inventory of Depressive SymptomatologySoSSocial Support ScaleTiC-PTreatment Inventory of Costs in patients with psychiatric disordersWIWhitely Index

#### Patient characteristics for trajectories of physical functioning

Between-trajectories differences in patient characteristics are presented in table 3. Participants in the different trajectories of physical functioning varied in all categories studied. Participants who improved markedly (‘poor physical functioning, marked improvement’) reported the lowest physical activity and highest personal control over illness at recruitment. In addition, they reported fewer symptoms and somatic comorbid diseases and higher social support than participants who remained ‘stable’ (‘poor physical functioning, stable’ trajectory). Compared with participants in the ‘moderate physical functioning, slight improvement’ trajectory, participants in both ‘poor’ trajectories (‘poor physical functioning, marked improvement’ and ‘poor physical functioning, stable’) reported more unfavourable cognitive, emotional and behavioural responses at recruitment. Participants in the least favourable trajectory (‘poor physical functioning, stable’) were older and suffered from more symptoms and comorbid diseases than participants in the ‘moderate physical functioning, slight improvement’ trajectory.

**Table 3:** Differences in patient characteristics for trajectories of physical functioning

Physical functioning (PCS) trajectories				
Trajectory	(A) Poor physical functioning, marked improvement (n=25)	(B) Poor physical functioning, stable (n=102)	(C) Moderate physical functioning, slight improvement (n=167)	P value between clusters
**Personal background**				
Women, n (%)	18 (72.0)	82 (80.4)	125 (74.9)	p=0.498
Education level, n (%)				**p=0.003**
Lower education	9 (36.0)	46 (45.1)	41 (24.6	
Intermediate education	11 (44.0)	38 (37.3)	67 (40.1)	
Higher education	5 (20.0)	18 (17.6)	59 (35.3)	
Age in years, range 19–70, mean (SD)	47.2 (12.9)	49.9 (11.4)	44.8 (12.6)	B>C **(p=0.003)**
Potentially traumatic events in childhood (<16 years) (LEQ), median (IQR; total range)	0 (1; 0–4)	1 (2; 0–7)	0 (1; 0–6)	p=0.151
Perfectionism (MDPS), mean (SD)*	71.0 (21.9)	71.7 (22.1)	68.7 (23.1)	p=0.956
Neuroticism (NEO-FFI), mean (SD)*	33.7 (9.6)	34.8 (8.3)	31.0 (8.9)	B>C**(p=0.003)**
Extraversion (NEO-FFI), mean (SD)*	37.6 (6.2)	36.6 (6.6)	39.6 (6.3)	B<C **(p<0.001)**
**Social and environmental background**				
Marital status, n (%)				p=0.507
Married or cohabiting	17 (68.0)	58 (56.9)	104 (62.3)	
Other (single, divorced, widow)	8 (32.0)	44 (43.1)	63 (37.7)	
Social support (SoS), median (IQR; total range)	59 (17; 22–60)	54 (16; 15–60)	57 (9; 33–60)	**p=0.047**
**Illness stressors**				
Number of symptoms (PSQ), mean (SD)	22.4 (6.7)	28.0 (8.8)	19.0 (8.7)	B>A **(p=0.012)**; B>C **(p<0.001)**
Duration of symptoms in years, median (IQR; total range)	6 (12; 0.17–40)	8 (19; 0.06–62)	3 (11; 0.02–50)	**p=0.018**
**Comorbid diseases**				
Number of somatic comorbid diseases (TiC-P),median (IQR; total range)	1 (2; 0–4)	2 (2.5; 0–8)	1 (2; 0–6)	**p<0.001**
Number of psychiatric comorbid disorders (TiC-P), median (IQR; total range)	0 (1.5; 0–3)	0 (1; 0–3)	0 (1; 0–3)	p=0.529
**Cognitive responses**				
Fear-avoidance cognitions (CBRQ), mean (SD)	9.9 (5.1)	12.01 (4.6)	8.5 (4.2)	B>C **(p<0.001)**
Catastrophising cognitions (CBRQ), mean (SD)	7.5 (3.6)	7.1 (3.0)	4.4 (3.2)	A>C **(p<0.001)**; B>C **(p<0.001)**
Damage cognitions (CBRQ), mean (SD)	9.8 (3.4)	11.0 (3.2)	9.0 (3.6)	B>C **(p<0.001)**
Embarrassment avoidance cognitions (CBRQ), median (IQR; total range)	7 (10.5; 0–18)	8 (6; 0–22)	6 (8; 0–24)	**p<0.001**
Symptom focusing (CBRQ), mean (SD)	10.4 (5.3)	11.0 (4.6)	10.0 (5.5)	p=0.325
Somatosensory amplification (SSAS), mean (SD)	28.6 (6.3)	27.6 (6.4)	26.6 (6.0)	p=0.398
Identity (label of disease and associated symptoms, IPQ-brief), mean (SD)	8.6 (1.2)	8.0 (1.6)	6.4 (1.9)	A>C **(p<0.001)**;B>C **(p<0.001)**
Consequences (beliefs about illness effects and outcomes, IPQ-brief), median (IQR; total range)	9 (3; 3–10)	8 (3; 3–10)	7 (4; 0–10)	**p<0.001**
Timeline (expected duration of illness, IPQ-brief), median (IQR; total range)	10 (3.5; 4–10)	9.5 (2; 3–10)	7 (4; 0–10)	**p<0.001**
Personal control (over illness, IPQ-brief), mean (SD)	7.7 (2.3)	5.8 (3.0)	5.6 (2.7)	B<A **(p=0.008)**;C<A **(p=0.002)**
Treatment control (beliefs about treatment effect on illness, IPQ-brief), mean (SD)	4.4 (2.9)	4.4 (2.5)	4.3 (2.6)	p=1.00
Comprehensibility (extent to which patients believe to understand their illness, IPQ-brief), mean (SD)	3.9 (3.3)	3.9 (2.4)	4.2 (2.8)	p=0.912
**Emotional responses**				
Anxiety severity (BAI), median (IQR; total range)	11 (11; 1–35)	12.5 (12.75;1–51)	8 (10, 0–38)	**p<0.001**
Depression severity (QIDS-SR), mean (SD)	10.4 (5.4)	11.1 (4.9)	7.8 (4.6)	A>C **(p=0.032)**;B>C **(p<0.001)**
Positive affect (PANAS), mean (SD)	30.6 (10.0)	31.0 (7.6)	33.8 (8.1)	B<C **(p=0.019)**
Health anxiety (WI), mean (SD)	6.0 (2.5)	6.8 (2.6)	5.1 (2.7)	B>C **(p<0.001)**
Concern (about illness, IPQ-brief), mean (SD)	7.3 (2.6)	6.1 (2.8)	4.8 (2.7)	A>C **(p<0.001)**;B>C **(p<0.001)**
Emotions (extent to which illness affects mood, IPQ-brief), mean (SD)	7.2 (2.9)	6.8 (2.6)	5.7 (2.9)	B>C **(p=0.009)**;A>C **(p=0.046)**
**Behaviouralresponses**				
All-or-nothing behaviour (CBRQ), mean (SD)	8.7 (4.4)	9.1 (4.2)	7.0 (4.5)	B>C **(p>0.001)**
Avoidance/resting behaviour (CBRQ), mean (SD)	10.0 (5.7)	9.8 (4.6)	7.0 (4.5)	A>C **(p=0.012)**;B>C **(p<0.001)**
Physical activity (IPAQ, in METs), median (IQR; total range)	1097 (3139; 0–35 523)	1875 (4327; 0–63 312)	3534 (6923;0–42 846)	**p<0.001**

In bold p<0.05.

* As indicated in the method section, these characteristics were measured at T1 (6 months follow-up); differences between trajectories were tested with ANOVA and post hoc Bonferroni correction for mean values to control for multiple testing, with χ2 tests for proportions and Kruskal-Wallis test for median values. For ANOVA and post hoc Bonferroni correction, we report only the results for trajectories that showed significant differences.

ANOVAanalysis of varianceBAIBeck Anxiety InventoryCBRQCognitive Behavioural Response Questionnaire (higher scores indicating more negative cognitive and behavioural responses)IPAQInternational Physical Activity QuestionnaireIPQ-briefBrief illness Perception Questionnaire (higher scores indicating more negative illness perceptions)LEQLife Events QuestionnaireMDPSMultidimensional Perfectionism ScaleMETsMetabolic EquivalentsNEO-FFINEO personality questionnaire- Five Factor InventoryPANASsubscale of Positive and Negative Affect SchedulePCSPhysical Component SummaryPSQPhysical Symptom QuestionnaireQIDS-SRQuick Inventory of Depressive SymptomatologySoSSocial Support ScaleSSASSomatosensory Amplification ScaleTiC-PTreatment Inventory of Costs in patients with psychiatric disordersWIWhitely Index

#### Patient characteristics for trajectories of mental functioning

The results of the analyses are shown in table 4. Patients with different trajectories of mental functioning also varied in all studied categories. In the ‘poor mental functioning, marked improvement’ trajectory, participants scored lowest on physical activity and reported more psychiatric comorbid disorders and a higher anxiety level at recruitment. Participants in the ‘deterioration’ trajectory scored lowest on social support. Participants in both the ‘marked improvement’ and ‘deterioration’ trajectories reported more symptoms and scored less favourable in terms of personality traits and cognitive, emotional and behavioural responses than participants in the ‘moderate mental functioning, slight improvement’ trajectory.

**Table 4:** Differences in patient characteristics for trajectories of mental functioning

Mental functioning (MCS) trajectories				
Trajectory	(A) Poor mental functioning marked improvement (n=41)	(B) Moderate mental functioning, deterioration (n=36)	(C) Moderate mental functioning, slight improvement (n=217)	P value between clusters
**Personal background**				
Women, n (%)	32 (78.0)	27 (75.0)	166 (76.5)	p=0.951
Education level, n (%)				p=0.577
Lower education	15 (36.6)	14 (38.9)	67 (30.9)	
Intermediate education	17 (41.5)	15 (41.7)	84 (38.7)	
Higher education	9 (22.0)	7 (19.4)	66 (30.4)	
Age in years, range 19–70, mean (SD)	47.0 (11.8)	46.0 (11.1)	46.8 (12.7)	p=1.00
Potentially traumatic events in childhood (<16 years)(LEQ), median (IQR; total range)	0 (2; 0–5)	0 (1; 0–7)	0 (1; 0–6)	p=0.824
Perfectionism (MDPS), mean (SD)*	81.1 (23.9)	81.7 (22.1)	66.0 (21.1)	B>C **(p<0.001)**; A>C **(p<0.001)**
Neuroticism (NEO-FFI), mean (SD)*	40.0 (7.8)	37.3 (6.4)	30.5 (8.5)	B>C **(p<0.001)**; A>C **(p<0.001)**
Extraversion (NEO-FFI), mean (SD)*	35.9 (6.9)	35.4 (6.4)	39.4 (6.2)	B<C **(p=0.004)**; A<C **(p=0.006)**
**Social and environmental background**				
Marital status, n (%)				p=0.397
Married or cohabiting	23 (56.1)	19 (52.8)	137 (63.1)	
Other (single, divorced, widow)	18 (43.9)	17 (47.2)	80 (36.9)	
Social support (SoS), median (IQR; total range)	55 (18; 22–60)	52.5 (15.5; 34–60)	57 (11; 15–60)	**p=0.027**
**Illness stressors**				
Number of symptoms (PSQ), mean (SD)	25.9 (8.8)	26.3 (9.5)	21.1 (9.4)	B>C **(p=0.008)**; A>C **(p=0.010)**
Duration of symptoms in years, median (IQR; total range)	6 (13; 0.42–30)	7 (16; 0.25–45)	4 (14.5; 0.02–62)	p=0.235
**Comorbid diseases**				
Number of somatic comorbid diseases (TiC-P),median (IQR; total range)	1 (2; 0–7)	1 (2.75; 0–6)	1 (2; 0–8)	p=0.799
Number of psychiatric comorbid disorders (TiC-P), median (IQR; total range)	1 (2; 0–3)	0 (1; 0–3)	0 (1; 0–3)	**p<0.001**
**Cognitive responses**				
Fear-avoidance cognitions (CBRQ), mean (SD)	10.2 (5.8)	11.9 (4.5)	9.5 (4.4)	B>C **(p=0.014)**
Catastrophising cognitions (CBRQ), mean (SD)	8.0 (3.6)	7.0 (3.4)	4.9 (3.1)	B>C **(0.002)**; A>C **(<0.001)**
Damage cognitions (CBRQ), mean (SD)	10.3 (3.6)	10.9 (3.9)	9.4 (3.4)	p=0.086
Embarrassment avoidance cognitions (CBRQ), median (IQR; total range)	11 (7.5; 0–24)	10 (5.5; 0–22)	6 (7.25; 0–18)	**p<0.001**
Symptom focusing (CBRQ), mean (SD)	13.1 (6.0)	11.5 (4.5)	9.6 (4.9)	A>C **(p<0.001)**
Somatosensory amplification (SSAS), mean (SD)	29.5 (6.9)	28.4 (4.8)	26.5 (6.1)	A>C **(p=0.013)**
Identity (label of disease and associated symptoms, IPQ-brief), mean (SD)	8.2 (1.7)	7.5 (1.7)	6.9 (2.0)	A>C **(p<0.001)**
Consequences (beliefs about illness effects and outcomes, IPQ-brief), median (IQR; total range)	9 (2; 5–10)	8 (3.75; 2–10)	7 (4; 0–10)	**p<0.001**
Timeline (expected duration of illness, IPQ-brief), median (IQR; total range)	9 (3; 3–10)	9 (3.75; 2–10)	8 (4; 0–10)	p=0.111
Personal control (over illness, IPQ-brief), mean (SD)	6.8 (3.3)	6.1 (2.7)	5.6 (2.7)	A>C **(p=0.040)**
Treatment control (beliefs about treatment effect on illness, IPQ-brief), mean (SD)	4.1 (2.9)	4.4 (2.8)	4.3 (2.5)	p=1.00
Comprehensibility (extent to which patients believe to understand their illness, IPQ-brief), mean (SD)	4.6 (3.3)	3.7 (2.5)	4.0 (2.6)	p=1.00
**Emotional responses**				
Anxiety severity (BAI), median (IQR; total range)	15 (14.5; 1–51)	12 (12.5; 1–44)_	9 (10; 0–42)	**p<0.001**
Depression severity (QIDS-SR), mean (SD)	13.2 (5.0)	10.9 (4.5)	8.1 (4.6)	B>C **(p=0.003)**; A>C **(p<0.001)**
Positive affect (PANAS), mean (SD)	26.3 (9.1)	30.2 (6.7)	34.1 (7.6)	B<C **(p=0.017)**; A<C **(p<0.001)**
Health anxiety (WI), mean (SD)	7.4 (2.8)	6.9 (2.4)	5.3 (2.6)	B>C **(p=0.002)**; A>C **(p<0.001)**
Concern (about illness, IPQ-brief), mean (SD)	7.0 (3.0)	6.2 (2.9)	5.1 (2.7)	A>C **(p<0.001)**
Emotions (extent to which illness affects mood, IPQ-brief), median (IQR; total range)	7.7 (2.5)	7.4 (1.9)	5.8 (2.9)	B>C **(p=0.003)**; A>C **(p<0.001)**
**Behaviouralresponses**				
All-or-nothing behaviour (CBRQ), mean (SD)	9.8 (4.3)	8.5 (4.4)	7.4 (4.4)	A>C **(p=0.004)**
Avoidance/resting behaviour (CBRQ), mean (SD)	11.7 (6.0)	9.9 (4.4)	7.3 (4.3)	B>C **(p=0.009)**; A>C **(p<0.001)**
Physical activity (IPAQ, in METs), median (IQR; total range)	1166 (4442; 0–63 312)	2696 (5627; 132–28 668)	2984 (6455; 0–44 436)	**p=0.046**

In bold p<0.05.

* As indicated in the method section, these characteristics were measured at T1 (6 months follow-up); differences between trajectories were tested with ANOVA and post hoc Bonferroni correction for mean values to control for multiple testing, with χ2 tests for proportions and Kruskal-Wallis test for median values. For ANOVA and post hoc Bonferroni correction, we report only the results for trajectories that showed significant differences.

ANOVAanalysis of varianceBAIBeck Anxiety InventoryCBRQCognitive Behavioural Response Questionnaire (higher scores indicating more negative cognitive and behavioural responses)IPAQInternational Physical Activity QuestionnaireIPQ-briefBrief Illness Perception Questionnaire (higher scores indicating more negative illness perceptions)LEQLife Events QuestionnaireMCSMental Component SummaryMDPSMultidimensional Perfectionism ScaleMETsMetabolic EquivalentsNEO-FFINEO Personality Questionnaire-Five Factor InventoryPANASsubscale of Positive and Negative Affect SchedulePSQPhysical Symptom QuestionnaireQIDS-SRQuick Inventory of Depressive SymptomatologySoSSocial Support ScaleSSASSomatosensory Amplification ScaleTiC-PTreatment Inventory of Costs in patients with psychiatric disordersWIWhitely Index

## Discussion

We identified distinct 5-year trajectories for symptom severity, physical and mental functioning in adult patients with PSS. For symptom severity, we identified two ‘stable’ trajectories, indicating a high prevalence of persisting symptoms in this population. Physical and mental functioning over the 5-year period improved slightly for the large majority of the participants. In a minority of participants, we identified trajectories that showed considerable improvement in physical or mental functioning (8.5%, respectively, 13.9% of participants), as well as a trajectory for deteriorating mental functioning (12.2% of participants). As anticipated, we found high inter-individual and intra-individual variation within the identified trajectories. A wide range of patient characteristics—such as personal, social and environmental background, illness stressors, comorbid diseases and cognitive, emotional and behavioural responses—varied between the distinct trajectories for symptom severity, physical and mental functioning.

This is the first study that applied LCGMM to study 5-year trajectories of symptom severity, physical and mental functioning in patients with PSS. Strengths of this study include its multicentre prospective design, the long duration of follow-up using multiple measurements over time, the low loss to follow-up and the use of LCGMM—a contemporary and flexible data-driven technique to distinguish distinct trajectories over time in a study population. We did not only focus on symptom persistence, but also on trajectories of physical and mental functioning: important aspects of prognosis in patients with PSS. An important limitation is the fact that trajectories identified by LCGMM represent a simplified version of the complex heterogeneous reality. Though the different trajectories found may be indicative of long-term general trends and summarise these adequately, individual trajectories can be more variable and diverse, with exacerbations and remissions that remain undetected by the LCGMM. Another limitation concerns the possibility of selection and attrition bias. The majority of participants in the PROSPECTS study already experienced symptoms for an extensive period of time when recruited (median duration 5 years; IQR 14 years), indicating that we may have selected more patients with long-lasting PSS by our inclusion procedure. Therefore, we should keep in mind that trajectories identified in this study may differ from trajectories shortly after onset of PSS. An additional limitation concerns the small number of participants in some of the trajectories; due to these small numbers, prediction modelling was not possible within our study, and examining differences in patient characteristics between trajectories was also hampered by the small subgroups. Some attrition bias may have occurred as well, though the study sample for the LCGMM consisted of over 90% of the initially recruited participants in the PROSPECTS study. Furthermore, the moment at which participants completed the questionnaire may have been influenced by the perceived severity of symptoms and their functional abilities, which in turn may affect our findings.

In most previous studies on the course of PSS, the majority of patients showed symptom improvement over time, and in a minority of patients, symptoms worsened or became chronic.^9^,^1371^ Our findings show a different picture: when using multiple measurements and applying LCGMM over a 5-year period, participants in the PROSPECTS cohort followed ‘stable’ trajectories for symptom severity, indicating a higher prevalence of symptom persistence than expected based on these previous studies. Our findings were largely consistent with our previous results on the 2-year course of PSS.^23^ The frequent occurrence of ‘slight improvement’ rather than ‘stable’ trajectories for physical and mental functioning in the current 5-year analyses indicates either a genuine adjustment leading to better functioning or a response shift leading to different reporting of functioning. A response shift can occur when individuals learn to adapt to their changed functioning and tend to report scores differently over time.^75^ The inter-individual and intra-individual heterogeneity within trajectories, was also present in our current analyses. Patterns of remissions and exacerbations of symptoms were confirmed in a qualitative study conducted among participants of the PROSPECTS study and were an important element of their symptom experience.^76^ Patterns of fluctuating symptoms have been described in patients with various long-term or chronic conditions, such as osteoarthritis,^77 78^ fibromyalgia^79 80^ and long COVID-19 symptoms.^81^

Patients in the distinct trajectories differed with regard to a wide range of patient characteristics previously associated with an (un)favourable prognosis of PSS. In the interpretation of our findings, we have to be aware that these analyses were primarily explorative in nature. However, our findings are largely consistent with previous studies and the theoretical background of (un)favourable prognostic factors in PSS.^10^,^21^ Of note is that participants who improved markedly in mental functioning, reported more unfavourable characteristics at baseline (eg, higher anxiety levels, more psychiatric comorbid disorders) than those in the other mental functioning trajectories. This finding may underline the importance of timely diagnosing psychiatric comorbid disorders, although it may also mean that this subgroup had more room for improvement (‘regression to the mean’) or was more receptive to treatment.

As indicated in the introduction, patients can suffer from PSS in the context of well-understood (adequately treated) conditions or when no somatic explanation for symptoms is found. Though explorative in nature, our findings on patient characteristics for the distinct trajectories may add to this current concept of PSS. For example, participants in the ‘severe symptom’ and ‘poor physical functioning’ trajectory reported more somatic comorbid diseases and lower social support. In addition, we found differences in cognitive, emotional and behavioural responses to symptoms for a range of trajectories, whereas the number of psychiatric comorbid diseases did not differ. These findings suggest that biological, psychological and social factors may all play a role in the persistence of somatic symptoms, irrespective of aetiology and question the outdated term and definition of ‘medically unexplained symptoms’.

The identified trajectories help to better understand long-term trajectories and prognosis of symptom severity, physical and mental functioning in adult patients who repeatedly visit their general practitioner or are referred to a specialised treatment facility for their PSS. Clinicians should be more aware of the high prevalence of persisting symptoms and limited improvement in physical and mental functioning with frequent exacerbations and remissions over time. We also identified trajectories of marked improvement in physical or mental functioning, as well as a trajectory of mental deterioration. Gaining more knowledge about these potentially clinically relevant subgroups may further assist clinical decision-making and evaluation. Future studies that follow patients shortly after the onset of their PSS and map their long-term course of PSS with multiple measurements over time and multiple outcomes are warranted. As are further predictive studies and studies on the identification of clinically relevant subgroups (as in the current study potentially relevant subgroups were small, for this purpose large study samples may be necessary). This may ultimately enable a more patient-centred approach to providing care for patients with PSS.

## Conclusion

We identified distinct 5-year trajectories for symptom severity, physical and mental functioning in adult patients with PSS. Our findings suggest, partly contrary to previous research, a high prevalence of persistence of symptoms and limited improvement in physical and mental functioning over the longer-term in a majority of adult patients with PSS. In a small proportion of patients, we identified trajectories that showed considerable improvement in physical or mental functioning, or deterioration in mental functioning. These may represent potentially clinically relevant subgroups of patients with PSS. A wide range of patient characteristics differed between the distinct trajectories, and these characteristics may help in early recognition, although predictive studies are warranted.

## supplementary material

10.1136/bmjopen-2023-083276 online supplemental file 1

10.1136/bmjopen-2023-083276 online supplemental file 2

10.1136/bmjopen-2023-083276 online supplemental file 3

## Data Availability

No data are available.
