# Supplementary material for: Five-year trajectories of symptom severity, physical and mental functioning in patients with persistent somatic symptoms: the PROSPECTS cohort study
Source: BMJ Open. 2025 Jan 8;15(1):e083276. doi: 10.1136/bmjopen-2023-083276 (PMC11749328; doi:10.1136/bmjopen-2023-083276)
Supplement: online supplemental file 1 [file bmjopen-15-1-s001.pdf]

Appendix A. Measures and time points of administration

	Instrument	Baseline (T0)	6 months (T1)	1 yr (T2)	2 yr (T3)	3yr (T4)	4yr (T5)	5yr (T6)
**Outcomes**								
Symptom severity	Patient Health Questionnaire-15 (PHQ-15) (30)	X	X	X	X	X	X	X
Physical functioning	RAND-36 Physical Component Summary (PCS) (34, 35)	X	X	X	X	X	X	X
Mental functioning	RAND-36 Mental Component Summary (MCS) (34, 35)	X	X	X	X	X	X	X
**Personal background**								
Sex, education level, age (in years)	Questionnaire	X						
Potentially traumatic events in childhood (<16 years)	Life Events Questionnaire (LEQ) (53)	X						
Perfectionism	Multi-dimensional Perfectionism Scale (MDPS) (54, 55))		X					
Neuroticism<br>Extraversion	Subscales NEO Personality questionnaire- Five Factor Inventory (NEO-FFI subscale) (56, 57)		X					
**Social and environmental background**								
Marital status	Questionnaire	X						
Social support	Social Support scale (SoS) (58)	X						
**Illness stressors**								
Number of symptoms	Physical Symptom Questionnaire (PSQ) (59)	X						
Duration of symptoms	Questionnaire	X						
**Comorbid diseases**								
Number of somatic comorbid diseases<br>Number of psychiatric comorbid disorders	Trimbos/iTMA questionnaire for Costs associated with Psychiatric Illness (TiC-P) (60)	X						
**Cognitive responses**								
Cognitions and coping	Cognitive behavioral responses questionnaire (CBRQ) (61, 62))	X						
Somatosensory amplification	Somatosensory amplification scale (SSAS) (63, 64)	X						
Illness perception (cognitive responses)	Illness Perception questionnaire-brief (IPQ-brief) (65, 66)	X						
**Emotional responses**								
Anxiety severity	Beck anxiety inventory (BAI) (67, 68)	X						
Depression severity	Quick inventory of depressive symptomatology (QIDS-SR) (69)	X						
Positive affect	Subscale of positive and negative affect schedule (PANAS) (70)	X						
Health anxiety	Whitely Index (WI) (64, 71)	X						
illness perception (emotional responses)	Illness Perception questionnaire-brief (IPQ-brief) (65, 66)	X						
**Behavioral responses**								
Behavioral responses to symptoms	CBRQ (61, 62)	X						
Physical activity	International Physical Activity Questionnaire (IPAQ) (72, 73)	X						
