# Supplementary material for: Five-year trajectories of symptom severity, physical and mental functioning in patients with persistent somatic symptoms: the PROSPECTS cohort study
Source: BMJ Open. 2025 Jan 8;15(1):e083276. doi: 10.1136/bmjopen-2023-083276 (PMC11749328; doi:10.1136/bmjopen-2023-083276)
Supplement: online supplemental file 2 [file bmjopen-15-1-s002.pdf]

Appendix B. Model fit indices

Model fit indices of the LCGMM results for symptom severity (PHQ-15) trajectories (selected model highlighted)

PHQ-15 quadratic models, estimated within-class intercept variance					
	BIC	AIC	N	Posterior probabilties	Entropy index
1 trajectory	10226.168	10185.537	297	1	N/A
2 trajectories	***10213.966***	***10158.560***	***250/ 47***	***0.618/ 0.956***	***0.616***
3 trajectories	10221.903	10151.722	37/ 217/ 43	0.495/ 0.908/ 0.650	0.537
4 trajectories	10235.658	10150.702	38/ 209/ 48/ 2	0.506/ 0.900/ 0.660/ 0.703	0.624
5 trajectories	10250.317	10150.586	51/ 3/ 41/ 200/ 2	0.673/ 0.839/ 0.538/ 0.886/ 0.730	0.673
6 trajectories	10273.091	10158.586	2/ 200/ 3/ 0/ 51/ 41	0.730/ 0.886/ 0.839/ 0.000/ 0.673/ 0.538	0.706

PHQ-15 linear models, estimated within-class intercept variance					
	BIC	AIC	N	Posterior probabilties	Entropy index
1 trajectory	10237.002	10200.065	297	1	N/A
2 trajectories	10218.255	10170.237	49/ 248	0.629/ 0.953	0.612
3 trajectories	10221.440	10162.340	38/ 214/ 45	0.496/ 0.899/ 0.666	0.533
4 trajectories	10232.640	10162.459	207/ 48/ 4/ 38	0.894/ 0.668/ 0.678/ 0.509	0.621
5 trajectories	10247.704	10166.442	4/ 81/ 2/ 56/ 154	0.739/ 0.779/ 0.527/ 0.606/ 0.839	0.647
6 trajectories	10260.581	10168.238	48/ 9/ 35/ 95/ 4/ 106	0.638/ 0.587/ 0.729/ 0.720/ 0.904/ 0.872	0.666

Model fit indices of the LCGMM results for physical functioning (PCS) trajectories (selected model highlighted)

PCS quadratic models, estimated within-class intercept variance					
	BIC	AIC	N	Posterior probabilties	Entropy index
1 trajectory	15340.782	15381.301	294	1	N/A
2 trajectories	15330.237	15274.983	85/ 209	0.729/ 0.912	0.531
3 trajectories	***15324.694***	***15254.706***	***25/ 167/ 102***	***0.711/ 0.912/ 0.851***	***0.705***
4 trajectories	15323.353	15238.630	97/ 168/ 4/ 25	0.842/ 0.921/ 0.906/ 0.695	0.764
5 trajectories	15332.967	15233.510	23/ 164/ 90/ 14/ 3	0.714/ 0.920/ 0.830/ 0.560/ 0.995	0.763
6 trajectories	15345.925	15231.734	3/ 161/ 86/ 22/ 1/ 21	0.997/ 0.914/ 0.810/ 0.558/ 0.754/ 0.705	0.763

PCS linear models, estimated within-class intercept variance					
	BIC	AIC	N	Posterior probabilties	Entropy index
1 trajectory	15380.106	15343.270	294	1	N/A
2 trajectories	15332.156	15284.270	49/ 245	0.597/ 0.957	0.593
3 trajectories	15315.597	15256.660	25/ 169/ 100	0.701/ 0.910/ 0.849	0.702
4 trajectories	15313.451	15243.463	14/ 22/ 90/ 168	0.535/ 0.728/ 0.832/ 0.920	0.720
5 trajectories	15326.383	15245.344	18/ 158/ 91/ 14/ 13	0.369/ 0.900/ 0.835/ 0.533/ 0.701	0.668
6 trajectories	15336.318	15244.228	11/ 16/ 130/ 20/ 88/ 29	0.727/ 0.596/ 0.880/ 0.463/ 0.767/ 0.655	0.659

Table A.3: Model fit indices of the LCGMM results for mental functioning (MCS) trajectories (selected model highlighted)

**MCS quadratic models, estimated within-class intercept variance**					
	**BIC**	**AIC**	**N**	**Posterior probabilities**	**Entropy**
1 trajectory	15467.545	15427.025	294	1	N/A
2 trajectories	15428.983	15373.730	255/ 39	0.965/ 0.620	0.690
3 trajectories	***15399.421***	***15329.433***	***41/ 36/ 217***	***0.666/ 0.775/ 0.929***	***0.698***
4 trajectories	15403.467	15318.745	6/ 165/ 79/ 44	0.672/ 0.900/ 0.709/ 0.766	0.675
5 trajectories	15396.252	15296.796	48/ 11/ 4/ 162/ 69	0.785/ 0.662/ 0.730/ 0.910/ 0.771	0.746
6 trajectories	15396.939	15282.748	15/ 148/ 12/ 61/ 54/ 4	0.714/ 0.911/ 0.734/ 0.775/ 0.692/ 0.797	0.743

**MCS linear models, estimated within-class intercept variance**					
	**BIC**	**AIC**	**N**	**Posterior probabilities**	**Entropy**
1 trajectory	15462.546	15425.710	294	1	N/A
2 trajectories	15417.827	15369.941	253/ 41	0.961/ 0.639	0.690
3 trajectories	15392.127	15333.189	172/ 67/ 55	0.905/ 0.769/ 0.746	0.648
4 trajectories	15392.602	15322.614	161/ 59/ 4/ 70	0.892/ 0.767/ 0.615/ 0.736	0.684
5 trajectories	15392.732	15311.693	67/ 16/ 5/ 51/ 155	0.714/ 0.754/ 0.801/ 0.700/ 0.909	0.709
6 trajectories	15404.169	15312.079	53/ 153/ 14/ 1/ 4/ 69	0.721/ 0.910/ 0.649/ 0.574/ 0.709/ 0.716	0.733
