# Supplementary material for: Five-year trajectories of symptom severity, physical and mental functioning in patients with persistent somatic symptoms: the PROSPECTS cohort study
Source: BMJ Open. 2025 Jan 8;15(1):e083276. doi: 10.1136/bmjopen-2023-083276 (PMC11749328; doi:10.1136/bmjopen-2023-083276)

Appendix C. Visual evaluation of trajectories of individual participants

In this appendix trajectories of individual participants are shown for each outcome, specified per LCGMM trajectory. The thin colored lines are the individual trajectories. The thick black lines indicate the trajectories that resulted from the LCGMM analyses.

Symptom severity

'severe symptoms, stable' trajectory (n=47):

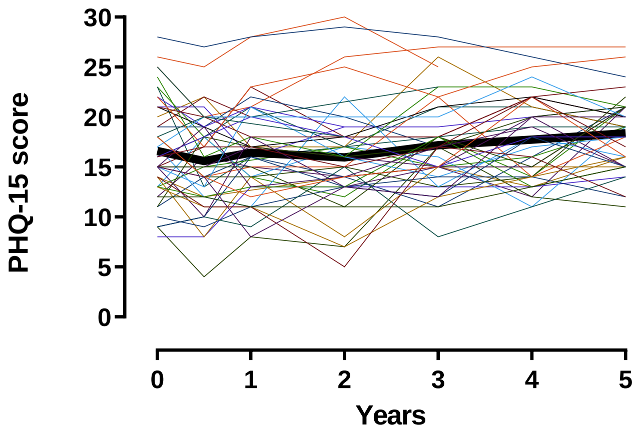

'moderate symptoms, stable' trajectory (n=250):

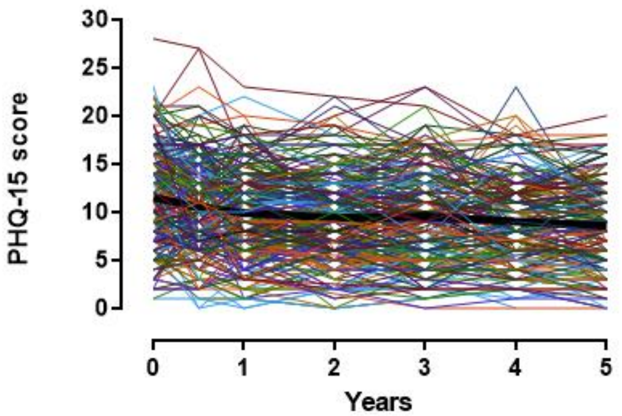

Physical functioning

'poor physical functioning, marked improvement' trajectory (n=25):

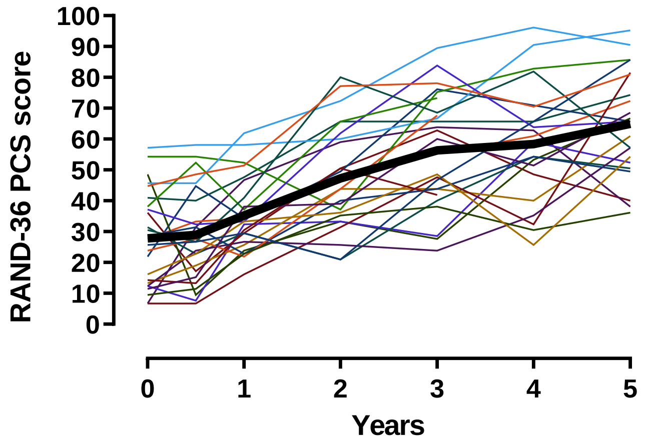

'poor physical functioning, stable' trajectory (n=102):

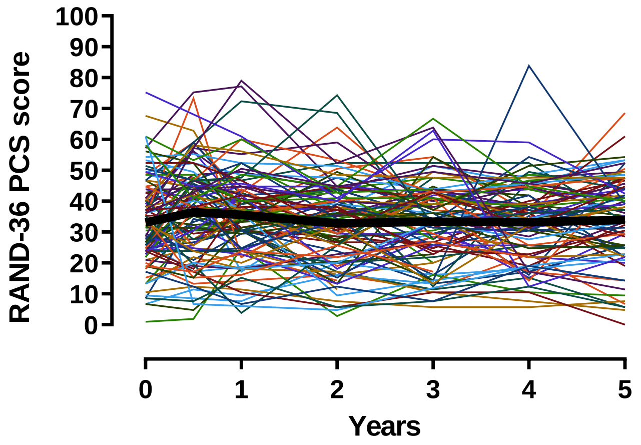

'moderate physical functioning, slight improvement' trajectory (n=167):

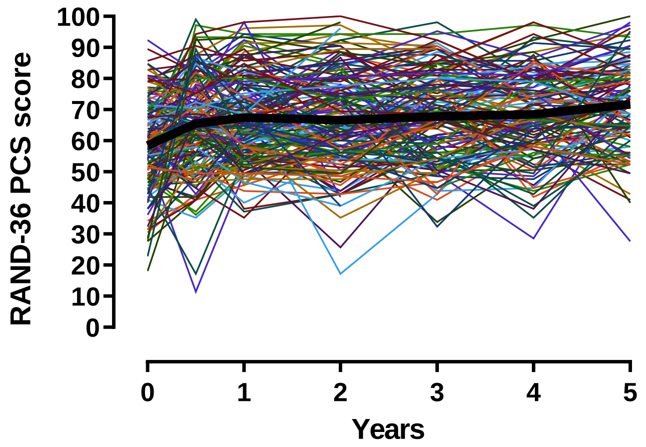

<u>**Mental functioning**</u>

‘poor mental functioning, marked improvement’ trajectory (n=41):

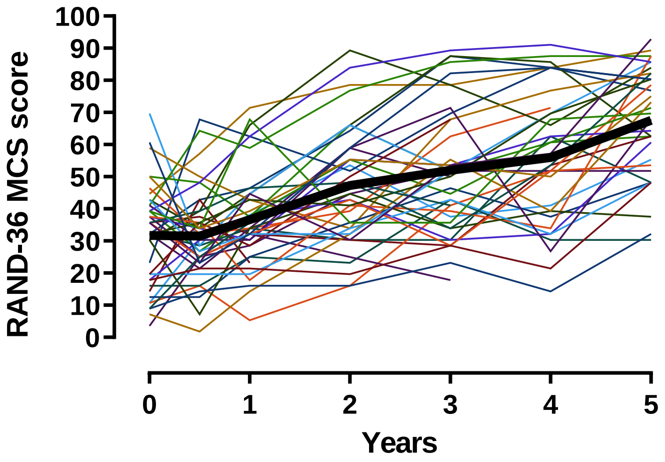

‘moderate mental functioning, deterioration’ trajectory (n=36):

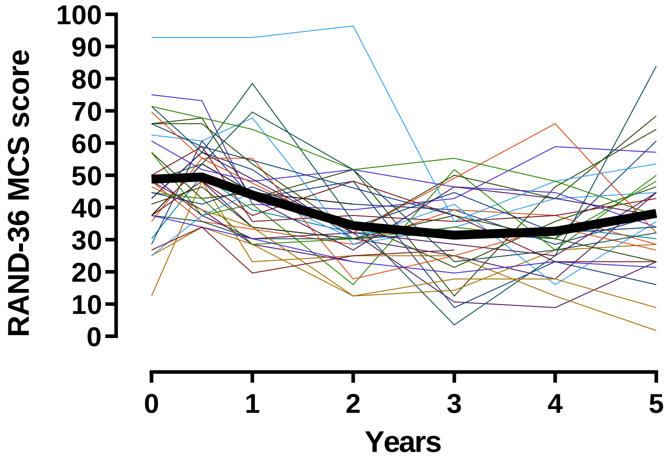

‘moderate mental functioning, slight improvement’ trajectory (n=217):

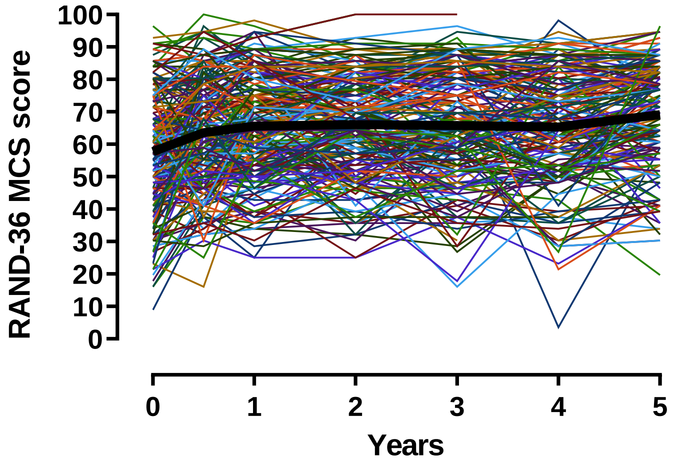

Supplement: online supplemental file 3 [file bmjopen-15-1-s003.pdf]
