# Supplementary material for: Five-year trajectories of symptom severity, physical and mental functioning in patients with persistent somatic symptoms: the PROSPECTS cohort study
Source: BMJ Open. 2025 Jan 8;15(1):e083276. doi: 10.1136/bmjopen-2023-083276 (PMC11749328; doi:10.1136/bmjopen-2023-083276)
Supplement: Supplementary file 4 [file bmjopen-2023-083276.reviewer_comments.pdf]

PEER REVIEW HISTORY

BMJ Open publishes all reviews undertaken for accepted manuscripts. Reviewers are asked to complete a checklist review form and are provided with free text boxes to elaborate on their assessment. These free text comments are reproduced below.

ARTICLE DETAILS

Title (Provisional)

Five-year trajectories of symptom severity, physical and mental functioning in patients with persistent somatic symptoms:the PROSPECTS cohort study

Authors

Barends, Hieke; van der Horst, Henriëtte E; van der Wouden, Johannes C.; Claassen, Nikki; Dekker, Joost; Hoekstra, Trynke

---

VERSION 1 - REVIEW

**Reviewer**	**1**
**Name**	**Alavi, Seyed Mohammad Amin**
**Affiliation**	**Ahvaz Jundishapur University of Medical Sciences**
**Date**	**21-Jan-2024**
**COI**	**Nothing to declare.**

Dear Editor,

Thank you for the opportunity to review the article entitled Five-year trajectories of symptom severity, physical and mental functioning in patients with persistent somatic symptoms: the PROSPECTS cohort study" for the Journal of BMJ Open.

The topic was fascinating, and I read the paper with great interest.

However, I would like to mention some points regarding the manuscript.

1. The definition of the PSS is repeated in the introduction and methods sections.

2. Please cite an article regarding the validity of RAND-36 (if available).

3. Please provide the information on the SPSS and Mplus software (company and location).

**Reviewer**	**2**
**Name**	**Kohlmann, Sebastian**

Affiliation **University Medical Center Hamburg-Eppendorf, Department of Psychosomatic Medicine and Psychotherapy**

Date **19-Feb-2024**

COI **none**

Short summary:

The manuscript reports on a longitudinal study investigating trajectories of somatic symptoms severity and quality of life in patients with persistent somatic symptoms (PSS). The results indicate that there are distinct trajectories of somatic symptom severity and quality of life over the course of 5 years. The authors conclude that somatic symptoms frequently persist over time and that there is little improvement in quality of life in this patient group.

General comment:

The manuscript is well-written in most parts and easy to follow (introduction and discussion); in other parts the manuscript would benefit from be more precise and present illustrations of the analyses (methods, results). The Latent Class Growth Mixed Modelling appears to be sound but should be reviewed by expert. Strengths of the study include its large dataset and long follow-up period. In fact, the manuscript reports on one of the longest follow-ups in somatic symptom research and could make an important contribution to the field of PSS. The manuscript would benefit from framing the rational within the context of the somatic symptom disorder diagnosis. Below, I have made some suggestions to improve the manuscript.

1. My main question is why did the authors choose quality of life as an outcome for trajectories? In the context of the relatively new diagnosis somatic symptoms disorder (SSD) it would be worthwhile to investigate the trajectories of health anxiety and constructs related to the b-criteria of SSD.

2. Throughout the manuscript the authors try to make a bridge between the old (outdated) term medically unexplained and persistent somatic symptoms. With respect to current concepts of PPS the manuscript would benefit presenting PSS irrespective of somatic aetiology (and omit all terms such as bio-medically explained). In fact, the results of this study indicate that the severe symptom trajectory is characterized by the number of somatic comorbidities which is an interesting finding in itself and undermines that PSS can be prevalent in patients with somatic diseases.

3. My second major concern is alpha-inflation. The analyses report on many t-tests with outcomes that are eventually highly correlated. I would advise to re-analyze and re-interpret the data using alpha-adjustments.

4. Even though details on the procedure have been published, for the reader it would be more informative to precise information on the assessment: where, when, who did the

assessments? Were the time points kept constant? If not, did variance in time have an impact on the trajectories?

5. I suppose that the analyses on the characteristics were conducted with parametric tests, but for some variables non-parametric tests would have been more adequate.

6. One questions that would be interesting is, whether patients in the trajectories overlap. It would very illustrative to present a figure that displays the overlap of somatic symptom and quality of life trajectories, or health anxiety trajectories?

7. As the authors note it would be interesting to identify predictors of unfavorable trajectories. However, currently I understand that nearly everyone in the sample stays on very burdened level. If this is the case, there is no variance to identify any groups. The authors may comment on this thought.

8. Patients were recruited in two different settings. Was setting a characteristic of the trajectories?

9. The discussion would benefit from interpreting relevant characteristics in the context of the current research on PSS, e.g., psychological burden differs but the number of mental comorbidities does not and numbers of somatic diseases differs, that questions the term 'medically unexplained'.

10. If the manuscript aims to make suggestions for the management of PSS, the authors should be very precise which of their results can inform current concepts of PSS care. Simply stating that PSS is frequent (is already known and) does not improve care. In my opinion, the manuscript does not need to make any assumptions about improving care, as the analyses are very exploratory and valuable in themselves.

11. Minor comment: Table 1: Duration of symptoms 'in years'?

VERSION 1 - AUTHOR RESPONSE

Reviewer: 1
Dr. Seyed Mohammad Amin Alavi, Ahvaz Jundishapur University of Medical Sciences
Comments to the Author:
Dear Editor,
Thank you for the opportunity to review the article entitled Five-year trajectories of symptom severity, physical and mental functioning in patients with persistent somatic symptoms: the PROSPECTS cohort study" for the Journal of BMJ Open.
The topic was fascinating, and I read the paper with great interest.
However, I would like to mention some points regarding the manuscript.
1. The definition of the PSS is repeated in the introduction and methods sections.

Response:

We thank the reviewer for reporting this, we have removed the definition from the methods section to avoid unnecessary repetition.

Changes:

We deleted the following sentence from the methods section (see p.6 'Main document – marked copy'): '*In the PROSPECTS study, PSS were defined as somatic symptoms that lasted at least several weeks and for which no sufficient somatic explanation was found after proper medical examination.'*

2. Please cite an article regarding the validity of RAND-36 (if available).

Response: Thank you for pointing this out, we refer to a reference that also discussed the validity of the (Dutch) RAND-36 in the methods section. We now have added this reference after stating that we selected the RAND-36 because of its validity (see p.9 of the 'Main document – marked copy').

3. Please provide the information on the SPSS and Mplus software (company and location).

Response:

Thank you for this addition, we have added additional information on the used software to the methods section.

Changes:

We added the following to the methods section (see p.6 'Main document – marked copy'):

IBM (New York) SPSS Statistics (Version 26)

Muthen & Muthen (Los Angeles) Mplus (Version 8.5)

Reviewer: 2
Dr. Sebastian Kohlmann, University Medical Center Hamburg-Eppendorf
Comments to the Author:

Short summary:
The manuscript reports on a longitudinal study investigating trajectories of somatic symptoms severity and quality of life in patients with persistent somatic symptoms (PSS). The results indicate that there are distinct trajectories of somatic symptom severity and quality of life over the course of 5 years. The authors conclude that somatic symptoms frequently persist over time and that there is little improvement in quality of life in this patient group.

General comment:
The manuscript is well-written in most parts and easy to follow (introduction and discussion); in other parts the manuscript would benefit from more precise and present illustrations of the analyses (methods, results). The Latent Class Growth Mixed Modelling appears to be sound but should be reviewed by expert. Strengths of the study include its large dataset and long follow-up period. In fact, the manuscript reports on one of the longest follow-ups in somatic symptom research and could make an important contribution to the field of PSS. The manuscript would benefit from framing the rational within the context of the somatic symptom disorder diagnosis.

Below, I have made some suggestions to improve the manuscript.

1. My main question is why did the authors choose quality of life as an outcome for trajectories? In the context of the relatively new diagnosis somatic symptoms disorder (SSD) it would be worthwhile to investigate the trajectories of health anxiety and constructs related to the b-criteria of SSD.

Response:

We thank the reviewer for posing this interesting question. When the PROSPECTS study was designed in 2013, we decided on the study objectives and main outcome measures. The main study objective was mapping the course of symptom severity and health related functioning over a long period of time (at first three years, later extended to five years of follow-up), the selected main outcome measures were therefore symptom severity (PHQ-15) and health related functioning/ quality of life (RAND-36). These questionnaires were selected based on their validity and previous use in this specific population (van Dessel et al 2014). Therefore, to facilitate the long-term follow-up and comparison between and within patients, we decided to use these two outcomes in the current study as well. These choices were therefore primarily guided by consistency arguments related to the original design of the PROSPECTS study (Claassen et al 2018).

Although we agree with the reviewer that it would be interesting to study trajectories of health anxiety and related constructs in patients with PSS, these questions are beyond the scope of this article.

References:

van Dessel N, Leone SS, van der Wouden JC, Dekker J, van der Horst HE. The PROSPECTS study: design of a prospective cohort study on prognosis and perpetuating factors of medically unexplained physical symptoms (MUPS). J Psychosom Res. 2014;76(3):200-6.

Claassen-van Dessel N, van der Wouden JC, Hoekstra T, Dekker J, van der Horst HE. The 2-year course of Medically Unexplained Physical Symptoms (MUPS) in terms of symptom severity and functional status: results of the PROSPECTS cohort study. J Psychosom Res. 2018;104:76-87.

2. Throughout the manuscript the authors try to make a bridge between the old

(outdated) term medically unexplained and persistent somatic symptoms. With respect to current concepts of PPS the manuscript would benefit presenting PSS irrespective of somatic aetiology (and omit all terms such as bio-medically explained). In fact, the results of this study indicate that the severe symptom trajectory is characterized by the number of somatic comorbidities which is an interesting finding in itself and undermines that PSS can be prevalent in patients with somatic diseases.

Response:

Thank you for this remark. As stated above, the PROSPECTS study was designed and started in 2013. At that time the then-current term medically unexplained (physical) symptoms (MU(P)S) was used, which was defined as *'somatic symptoms that last at least several weeks and for which no sufficient somatic explanation is found after proper medical examination by a physician'* was used. Meeting this definition was an inclusion criterion for participants included in the PROSPECTS study. We think it is important to correctly describe the original inclusion criteria and definition when describing the PROSPECTS-cohort but also to elaborate on why we choose to use the current term PSS in our manuscript. As we indicate in the manuscript, patients can suffer from PSS in the context of well-understood (adequately treated) conditions or when no somatic explanation for symptoms is found.

We agree with the reviewer that the results regarding e.g. the severe symptom trajectory (being characterized by the number of somatic comorbidities) adds to the discussion on the current concept of PSS and we decided to add more on this to the discussion section of our manuscript.

Changes:

We deleted the following from the introduction (Box 1), *(*see p.4 'Main document – marked copy'): *"the focus in this publication is on the latter."* And added *"Biological, psychological and social factors may all play a role in the persistence of somatic symptoms."*

We added the following to the discussion section *(*see p.26 'Main document – marked copy') (also see our reply under point 9)*:*

As indicated in the introduction, patients can suffer from PSS in the context of well-understood (adequately treated) conditions or when no somatic explanation for symptoms is found. Though explorative in nature, our findings on patient characteristics for the distinct trajectories may add to this current concept of PSS. E.g., participants in the 'severe symptom' and 'poor physical functioning' trajectory reported more somatic comorbid diseases and lower social support. In addition, we found differences in cognitive, emotional and behavioral responses to symptoms for a range of trajectories, whereas the number of psychiatric comorbid diseases did not differ. These findings suggest that biological, psychological and social factors may all play a role in the persistence of somatic symptoms, irrespective of etiology and question the outdated term and definition of 'medically unexplained symptoms'

3. My second major concern is alpha-inflation. The analyses report on many t-tests with outcomes that are eventually highly correlated. I would advise to re-analyze and re-interpret the data using alpha-adjustments.

Response:

We thank the reviewer for mentioning this concern. Our second research objective was to *explore* patient characteristics associated with the trajectories. So, these analyses were primarily explorative in nature, and so is our interpretation of the results. In that respect, we indeed realize the possibility of alpha-inflation and took care in interpreting the statistical significance of the findings. Related to that, we describe that we used post-hoc Bonferroni correction, we did this to control for the overall probability of Type I error (false positive) for multiple hypothesis testing. We realize that this may not have been stated clear enough and have therefore added the reason why we used the post-hoc Bonferroni correction to the methods section and underlined this more in Table 3 and 4. Additionally, we attempted to place a stronger focus on the exploratory focus of this study in both the methods and discussion section of the manuscript.

Changes:

Methods section, see p. 12 of the 'Main document – marked copy':

We slightly altered the following sentence:

…and finally we ~~analyzed~~ explored differences in the selected patient characteristics between the identified trajectories (step 3)

and added:

Post hoc Bonferroni correction was applied to control for the overall probability of Type I error (false positive) for multiple hypothesis testing.

(Table 3 and 4, see p. 21 and 23): we added 'to control for multiple testing' to the sub text of the Tables

Discussion section, see p.25 of the 'Main document – marked copy':

We added the highlighted sentence and slightly altered the following paragraph:

Patients in the distinct trajectories differed with regard to a wide range of patient characteristics previously associated with an (un)favorable prognosis of PSS. In the interpretation of our findings we have to be aware that these analyses were primarily explorative in nature. However, our findings are largely consistent with previous studies and theoretical background of (un)favorable prognostic factors in PSS (14-25).

4. Even though details on the procedure have been published, for the reader it would be more informative to precise information on the assessment: where, when, who did the

assessments? Were the time points kept constant? If not, did variance in time have an impact on the trajectories?

Response:

Thank you for pointing this out, we agree with the reviewer that it is informative for the reader to receive more information on the assessment and decided to add this to the methods section of our manuscript.

Changes:

*We added the following (*see p.7 'Main document – marked copy')*: "The questionnaires were sent by mail. The time intervals were kept constant. After the baseline measurement, follow-up measurements took place at 6 months, 1, 2, 3, 4 and 5 years."*

5. I suppose that the analyses on the characteristics were conducted with parametric tests, but for some variables non-parametric tests would have been more adequate.

Response:

Indeed, the statistical techniques used depend on the distribution of the data and analyzed accordingly. Normally distributed data were tested with parametric tests; non-parametric tests were used for non-normally distributed data. We added additional information about this to the methods section of our manuscript to make this clearer.

Changes:

*We replaced (*see p.12 'Main document – marked copy')

"For categorical variables, we used χ2 tests. For continuous variables we used either independent sample t tests or ANOVA and post hoc Bonferroni correction, depending on the number of trajectories identified. For continuous variables with skewed distribution, we used Mann-Whitney test or Kruskal-Wallis test, depending on the number of trajectories."

By:

"Normally distributed data were tested with parametric tests; non-parametric tests were used for non-normally distributed data. For categorical variables, we used χ2 tests. For continuous variables with a normal distribution we used either the (parametric) independent sample t tests or ANOVA and post hoc Bonferroni correction, depending on the number of trajectories identified. For continuous variables with skewed distribution, we used the (non-parametric) Mann-Whitney test or Kruskal-Wallis test, depending on the number of trajectories."

6. One questions that would be interesting is, whether patients in the trajectories overlap. It would very illustrative to present a figure that displays the overlap of somatic symptom and quality of life trajectories, or health anxiety trajectories?

Response: Thank you for this question. In fact, we considered this additional question in an earlier phase of our study, but due to the number of trajectories, it was difficult to put into

figures and showed to be confusing rather than clarifying. Therefore, we decided not to include this additional question on overlap in trajectories in the final manuscript. Below and on the next pages we do include tables illustrating the overlap between trajectories for the Reviewer's interest.

Overlap of trajectories of physical functioning and symptom severity

		Physical functioning (RAND-36 PCS) trajectories			
		Poor physical functioning, marked improvement	Moderate physical functioning, slight improvement	Poor physical functioning, stable	Total
Symptom severity (PHQ-15) trajectories	Severe symptoms, stable	2	9	36	47
	Moderate symptoms, stable	23	158	66	247
	total	25	167	102	294

Overlap of trajectories of mental functioning and symptom severity

		Mental functioning (RAND-36 MCS) trajectories			
		Poor mental functioning, marked improvement	Moderate mental functioning, deterioration	Moderate mental functioning, slight improvement	Total
Symptom severity (PHQ-15) trajectories	Severe symptoms, stable	7	14	26	47
	Moderate symptoms, stable	34	22	191	247
	total	41	36	217	294

Overlap of trajectories of mental functioning and physical functioning

		Mental functioning (RAND-36 MCS) trajectories			
		Poor mental functioning, marked improvement	Moderate mental functioning, deterioration	Moderate mental functioning, slight improvement	Total
Physical functioning (RAND-36 PCS) trajectories	Poor physical functioning, marked improvement	14	0	11	25
	Moderate physical functioning, slight improvement	13	5	149	167
	Poor physical functioning, stable	14	31	57	102
	total	41	36	217	294

7. As the authors note it would be interesting to identify predictors of unfavorable trajectories. However, currently I understand that nearly everyone in the sample stays on very burdened level. If this is the case, there is no variance to identify any groups. The authors may comment on this thought.

Response:

Thanks for sharing this thought, let us indeed comment on this. Though we agree with the reviewer that the large majority remains (nearly) on the same burdened level, we also do see that there are indeed subgroups of participants (albeit small) who e.g. remain very low functioning (being a -relatively- unfavorable trajectory) as compared to others who improve significantly in functioning (being a -relatively- favorable trajectory).

We decided to add to the discussion more on the sample size of the trajectories and its impact on prediction.

Changes: We added the following to the discussion section (see p.24-25 'Main document – marked copy')

Another limitation concerns the small number of participants in some of the trajectories; due to these small numbers, prediction modeling was not possible within our study, and examining differences in patient characteristics between trajectories was also hampered by the small subgroups.

and p.26 'Main document – marked copy':

(as in the current study potentially relevant subgroups were small, for this purpose large study samples may be necessary).

8. Patients were recruited in two different settings. Was setting a characteristic of the trajectories?

Response: The recruitment setting did not differ significantly between trajectories ($\chi 2$ tests, see table on next page). Throughout the manuscript, we decided not to highlight the recruitment setting as patients may have changed setting over time (although we did not monitor this).

	**Included in primary care**	**Included in specialized PSS care**	**Total**
**Symptom trajectories (p= 0.311)**			
Severe symptoms, stable (N,%)	28 (14.3%)	19 (18.8%)	47
Moderate symptoms, stable (N,%)	168 (85.7%)	82 (81.2%)	250
total	196 (100%)	101 (100%)	297
**Physical functioning trajectories (p=0.066)**			
Poor physical functioning, stable	59 (30.6%)	43 (42.6%)	102
Moderate physical functioning, slight improvement	119 (61.7%)	48 (47.5%)	167
Poor physical functioning, marked improvement	15 (7.8%)	10 (9.9%)	25
Total	193	101	294
**Mental functioning trajectories (p=0.095)**			
Moderate mental functioning, deterioration	23 (11.9%)	13 (12.9%)	36
Moderate mental functioning, slight improvement	149 (77.2%)	68 (67.3%)	217
Poor mental functioning, marked improvement	21 (10.9%)	20 (19.8%)	41
	193	101	294

9. The discussion would benefit from interpreting relevant characteristics in the context of the current research on PSS, e.g., psychological burden differs but the number of mental comorbidities does not and numbers of somatic diseases differs, that questions the term 'medically unexplained'.

Response: Thank you for your helpful suggestion. We agree that (in addition to the reviewers comment under point 2), this is certainly an important point to discuss and we have added it to the discussion section of our manuscript.

Changes:

We added the following to the discussion section *(*see p.26 'Main document – marked copy') (also see our reply under point 2)*:*

As indicated in the introduction, patients can suffer from PSS in the context of well-understood (adequately treated) conditions or when no somatic explanation for symptoms is found. Though explorative in nature, our findings on patient characteristics for the distinct trajectories may add to this current concept of PSS. E.g., participants in the 'severe symptom' and 'poor physical functioning' trajectory reported more somatic comorbid diseases and lower social support. In addition, we found differences in cognitive, emotional and behavioral responses to symptoms for a range of trajectories, whereas the number of psychiatric comorbid diseases did not differ. These findings suggest that biological, psychological and social factors may all play a role in the persistence of somatic symptoms, irrespective of etiology and question the outdated term and definition of 'medically unexplained symptoms'.

10. If the manuscript aims to make suggestions for the management of PSS, the authors should be very precise which of their results can inform current concepts of PSS care. Simply stating that PSS is frequent (is already known and) does not improve care. In my opinion, the manuscript does not need to make any assumptions about improving care, as the analyses are very exploratory and valuable in themselves.

Response: Thank you for this helpful feedback, we agree that we should be cautious about making assumptions about improving the management of care based on our findings and indeed, the analyses are exploratory and valuable in themselves. However, based on prior literature, it is not common knowledge that even over the course of five years, symptoms tend to persist with mostly limited improvement in physical and mental functioning.

As we emphasize throughout our manuscript, most previous studies on the course of PSS (based on two follow-up measurements) indicates that the majority of patients showed symptom improvement over time, and that only in a minority of patients did symptoms worsen or become chronic. Thus, the degree of persistence of symptoms is still often underestimated. We believe that for clinicians is important to take note of the high prevalence of this persistence, even over a five year period. However, indeed, this does not directly affect the management of PSS.

Changes:

We added the highlighted text tot the discussion (conclusion) section (see p.26 'Main document – marked copy')

Our findings suggest, partly contrary to previous research, a high prevalence of persistence of symptoms and limited improvement in physical and mental functioning over the longer term in a majority of adult patients with PSS.

11. Minor comment: Table 1: Duration of symptoms 'in years'?

Response: Thank you for your observation, we have added 'in years' in Table 1, as well as Table 2, 3 and 4 (as it was also applicable to these Table 2-4).

Reviewer: 1
Competing interests of Reviewer: Nothing to declare.

Reviewer: 2
Competing interests of Reviewer: none

VERSION 2 - REVIEW

**Reviewer**	**2**
**Name**	**Kohlmann, Sebastian**
**Affiliation**	**University Medical Center Hamburg-Eppendorf, Department of Psychosomatic Medicine and Psychotherapy**
**Date**	**14-Nov-2024**
**COI**	

All my points have been adequately answered. I would like to thank the authors for their thorough revision.
